# Reduced Virulence and Enhanced Host Adaption during Antibiotics Therapy: a Story of a Within-Host Carbapenem-Resistant Klebsiella pneumoniae Sequence Type 11 Evolution in a Patient with a Serious Scrotal Abscess

**DOI:** 10.1128/msystems.01342-21

**Published:** 2022-03-01

**Authors:** Meiping Ye, Chunjie Liao, Mengya Shang, Danyang Zou, Xin Feng, Xinying Lu, Yixin Zhang, Jingmin Yan, Zhixiang Hu, Xiaogang Xu, Jianping Jiang, Pingyu Zhou

**Affiliations:** a STD Institute, Shanghai Skin Disease Hospital, Tongji University School of Medicine, Shanghai, China; b Shanghai Skin Disease Hospital, Clinical School of Anhui Medical University, Shanghai, China; c Institute of Antibiotics, Huashan Hospital, Fudan University, Shanghai, China; Southern Medical University

**Keywords:** RNA-seq, genomic study, host adaptation, *in vivo* evolution, virulence attenuation

## Abstract

Carbapenem-resistant Klebsiella pneumoniae (CRKP) has disseminated globally and threatened human life. The sequence type (ST) 11 CRKP is a dominant clone in Asia, but how this clone evolves *in vivo* then adapts to the host and facilitates dissemination remains largely unknown. Here, the genomic dynamics of 4 ST11-CRKP isolates, which were sequentially collected from the urine of a patient with initial serious scrotal abscess and finally recovered without effective medication, were analyzed. Genomic differences were identified and their implications for pathogenesis and host adaptation were investigated. The related transcriptional pathways were further explored by RNA-Seq. Genomic analysis identified 4 to 24 mutations, among which 94% to 100% of them were synonymous or intergenic mutations. During 47 days of antibiotics therapy, CRKP underwent adaptive evolution, including tigecycline resistance and virulence attenuation. Tigecycline resistance was caused by a deletion within the *ramR* ribosomal binding site, which has been described by us previously. On the other hand, mutations associated with two genes, acyltransferase (*act*) and *ompK26*, resulted in the attenuation phenotype of ST11-CRKP. *act* deficiency reduced the capsular polysaccharide (CPS) production, enhanced biofilm formation, weakened capsular protection, and decreased induction of proinflammatory cytokines. Further RNA-Seq analysis revealed that *act* influenced the expression of *ldhA*, *bglX*, *mtnK*, and *metE* which likely participate in capsular synthesis and biofilm formation. *ompK26* affected the virulence by its overexpression caused by the deletion of the upstream repressor binding site. This study presents a within-host adaption of ST11-CRKP and suggests an important role of CPS in the adaptive evolution of virulence and persistence of CRKP.

**IMPORTANCE** Carbapenem-resistant Klebsiella pneumoniae (CRKP) has disseminated worldwide and can cause life-threatening infections, including pneumonia, bloodstream infections, urinary tract infections, intraabdominal infection, liver abscess, and meningitis. CRKP infection is the leading cause of high mortality in hospitals. The sequence type (ST) 11 CRKP is a dominant clone and accounts for 60% of CRKP infections in China. Recently, the ST11-CRKP with high transmissibility is increasingly identified. Understanding how this clone has evolved is crucial for developing strategies to control its further dissemination. The significance of our research is the identification of the *in vivo* genomic dynamics of ST11-CRKP and the genetic basis for ST11-CRKP that facilitate persistence and dissemination. Furthermore, our study also highlights the importance of monitoring the within-host evolution of pathogens during the treatment and developing interventions to minimize the potential impact of host adaptation on human health.

## INTRODUCTION

The worldwide dissemination of carbapenem-resistant *Enterobacteriaceae* (CRE) is an urgent threat to public health (1). The World Health Organization (WHO) has classified CRE as one of the highest priority pathogens (2). Within CRE, carbapenem-resistant Klebsiella pneumoniae (CRKP) is the most common genus. One study showed that CRKP infection accounts for 80% of CRE infection and is the leading cause of high mortality in clinical infections (3).

Most of the CRKP belong to the clonal group CG258, in which the sequence types (ST) 258 and ST11 are dominant (4). ST11-CRKP is a prevalent clone in Asia and accounts for 60% of CRKP in China (5). Most recently, a novel subclone of ST11-CRKP with higher transmissibility has been identified in China (6), posing a greater challenge for CRKP infection control and surveillance. Though with extensive interrogations, the genetic elements responsible for the successful dissemination of ST11-CRKP have not been fully identified. Comprehensive genotypic and phenotypic studies of ST11-CRKP have identified genetic factors promoting dissemination such as those involved in carbapenem resistance, transmissibility, pathogenicity, and biofilm formation (7, 8). Furthermore, within-host adaptation was found to facilitate clone transmission as well (9–11), which was further highlighted by a study of the within-patient evolution of an ST258-CRKP (12). Until now, less is understood about the within-host evolution and adaptation of ST11-CRKP, especially from the genomic and transcriptomic perspectives.

In this study, we analyzed the within-host evolution of ST11-CRKP with whole-genome sequencing (WGS). ST11-CRKP strains were isolated longitudinally during 47 days of tigecycline-containing antibiotic therapy from the urine samples of a patient with initial serious scrotal abscess and finally recovered without effective medication treatment. During this course, the ST11-CRKP has undergone a series of phenotypic variations including the development of tigecycline resistance, reduced capsular polysaccharide (CPS) production, enhanced biofilm formation, weakened capsular protection, and decreased proinflammatory cytokines induction, and then its adaptation to the host environment was increased as it symbioses with the patient. We identified the genomic changes that contribute to these phenotypic variations leading to virulence reduction while acquiring tigecycline resistance. This study reveals how genomic variations led to the within-host adaption of ST11-CRKP in patients during antibiotic therapy.

## RESULTS

### The virulence of CRKP was attenuated *in vivo* during the antibiotics therapy.

Four CRKP strains (KP-1S, KP-2S, KP-3R, and KP-4R) were isolated longitudinally during 47 days of tigecycline-containing antibiotics therapy from the urine samples of a 50-year-old male patient with a scrotal abscess. The initial isolates (KP-1S and KP-2S) were susceptible to tigecycline (with MIC of 2 mg/liter) and subsequent isolates (KP-3R and KP-4R) were resistant to tigecycline (with MIC of 8 mg/liter). All four isolates were resistant to 15 other antimicrobials, including imipenem, meropenem, cefepime, amikacin, ceftazidime, cefuroxime, aztreonam, ciprofloxacin, levofloxacin, cefazolin, ceftriaxone, trimethoprim-sulfamethoxazole, cefoperazone/sulbactam, piperacillin-tazobactam, and cefepime/tazobactam, except polymyxin B (Table S1). At that time, polymyxin B was not approved by National Medical Products Administration in China and no effective medication was available for the treatment.

10.1128/msystems.01342-21.1 TABLE S1 Antimicrobial susceptibility patterns of K. pneumoniae KP-1S, KP-2S, KP-3R and KP-4R. Download Table S1, XLSX file, 0.01 MB. Copyright © 2022 Ye et al. 2022 Ye et al. https://creativecommons.org/licenses/by/4.0/ This content is distributed under the terms of the Creative Commons Attribution 4.0 International license.

**FIG 1:**
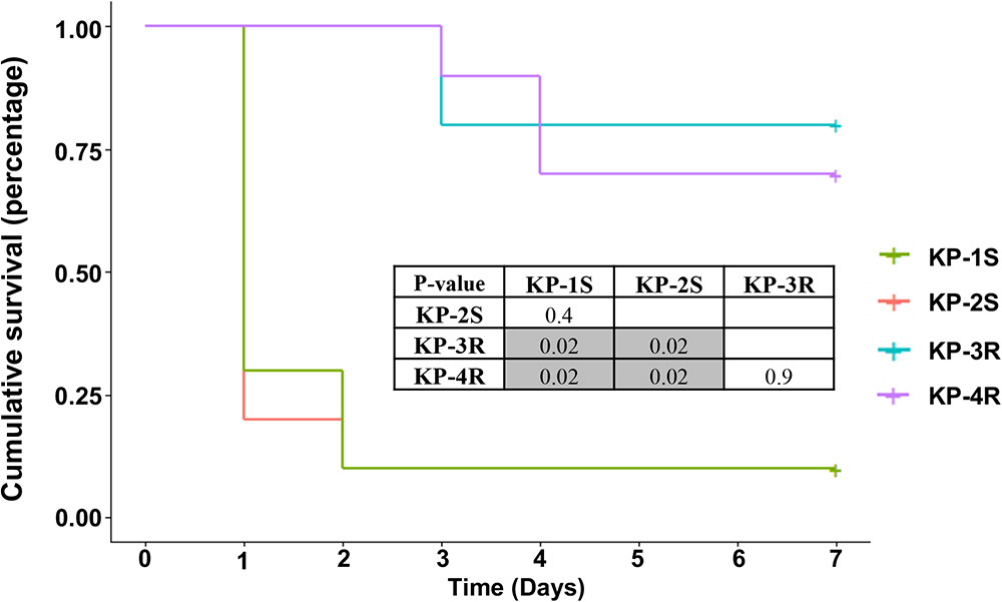
Kaplan-Meier survival curves of mice intraperitoneally challenged with Klebsiella pneumoniae ST11-CRKP isolates KP-1S, KP-2S, KP-3R, and KP-4R. Groups of 10 mice were inoculated with 10^6^ CFU and monitored daily for 7 days. *P* values were calculated using the Mantel-Cox log-rank test for survival curve comparison. Numbers in gray boxes indicate significant values (*P* < 0.05).

By the end of tigecycline-containing antibiotic therapy, the scrotal abscess condition of the patient stabilized and did not progress further. We hypothesized that CRKP might have evolved *in vivo* during the treatment, and the virulence of the longitudinally collected CRKP isolates has been reduced gradually. To test the hypothesis, groups of mice were inoculated with KP-1S, KP-2S, KP-3R, and KP-4R. As shown in Fig. 1, the mortality rate of mice inoculated with KP-1S or KP-2S was significantly higher than that of mice inoculated with KP-3R (90% versus 20%, *P* < 0.05; 90% versus 20%, *P* < 0.05) or KP-4R (90% versus 30%, *P* < 0.05; 90% versus 30%, *P* < 0.05), indicating that the virulence of KP-3R and KP-4R had attenuated compared to KP-1S and KP-2S.

### ST11-CRKP underwent limited genetic changes *in vivo*.

To identify the genetic element conferring the virulence attenuation, the genomes of the four isolates were subjected to whole-genome sequencing. Genomic analysis showed that all CRKP isolates belong to KL64-ST11, a prevalent subclone found in China. All isolates harbor carbapenemase-encoding gene *bla*_KPC-2_ and extended-spectrum β-lactamase-encoding gene *bla*_CTX-M-65_, which confers resistance to carbapenem and cephalosporin. They all possessed virulence genes, including yersiniabactin genes (*ybtAEPQSTUX*, *irp1*, *irp2*, and *fyuA*), aerobactin genes (*iutA* and *iucABCD*), *rmpA,* and *rmpA2* (Table 1). The aerobactin genes, *rmpA* and *rmpA2*, are located on the hypervirulent plasmid and yersiniabactin genes were located on the chromosome in hypervirulent K. pneumoniae (hvKP) NTUH-K2044. Plasmid replicons, including ColRNAI, IncFIB(K), IncFII, IncHI1B, and IncR, were present in all the isolates. Among them, IncFIB(K) and IncHI1B were associated with the hypervirulent plasmid in hvKP, and IncFII and IncR were usually associated with *bla*_KPC-2_ in CRKP.

**TABLE 1:** Genomic features of KP-1S, KP-2S, KP-3R, and KP-4R

Isolates	MLST	KPC	ESBL	*wzi*	Yersiniabactin	Aerobactin	*rmpA*	*rmpA2*	Plasmid inc
KP-1S, KP-2S, KP-3R and KP-4R	ST11	KPC-2	CTX-M-65	wzi64	ybt 9 (ICEKp3)	iuc 1	rmpA-2 (KpVP-1)	rmpA2-3 (−47%)	ColRNAI, IncFIB(K), IncFII, IncHI1B, IncR

**FIG 2:**
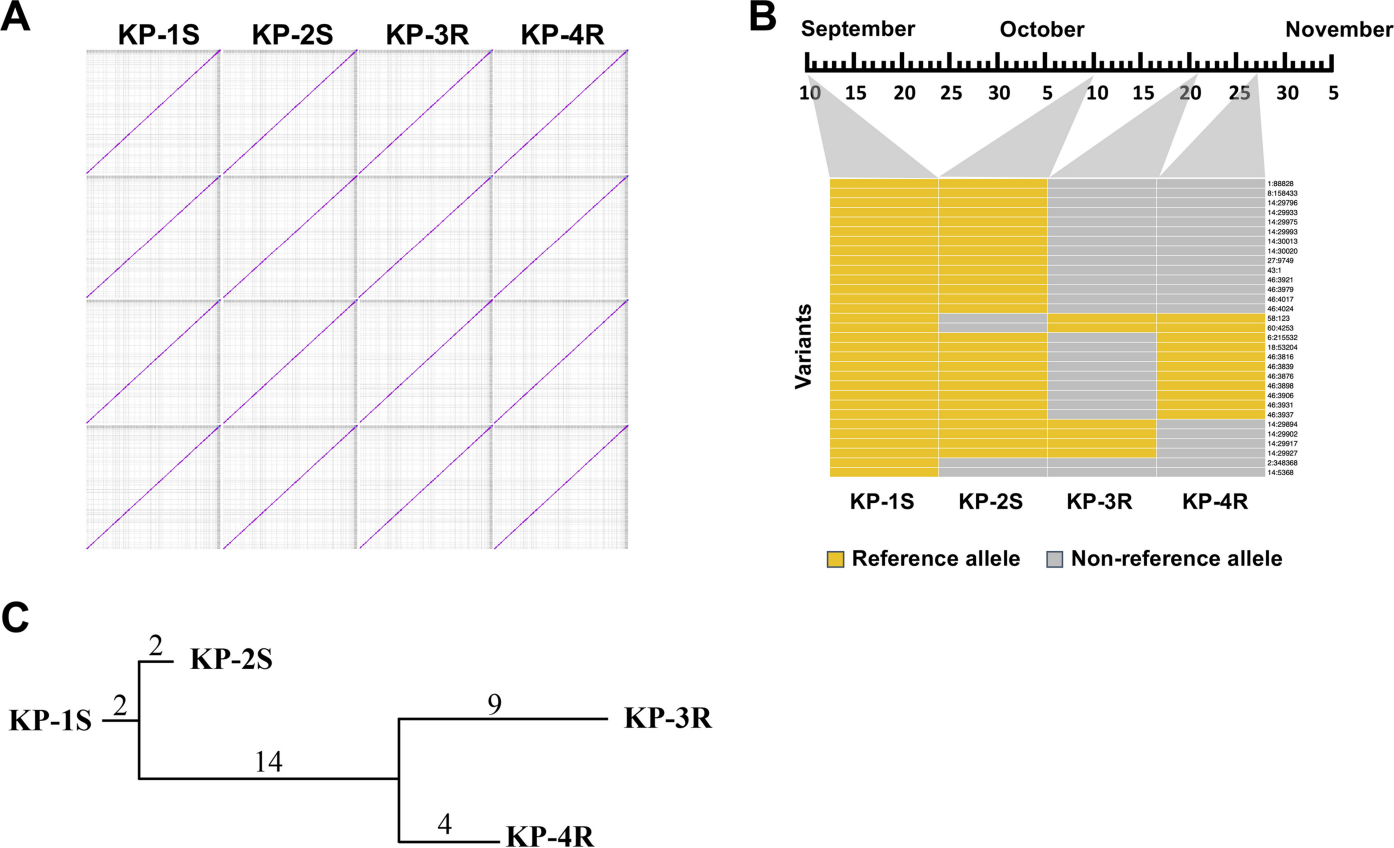
Genomic analysis of KP-1S, KP-2S, KP-3R, and KP-4R. (A) The pairwise genome alignments of KP-1S, KP-2S, KP-3R, and KP-4R. (B) Genomic variants of each ST11-CRKP isolates. (C) The phylogenetic tree of four ST11-CRKP isolates is based on the genomic variants. Numbers labeled above the line are the number of mutations. Mutation rates were calculated based on the number of variations and isolation timespan.

Whole-genome alignments showed that no genomic rearrangement was detected in KP-2S, KP-3R, or KP-4R compared to the genome structure of KP-1S, indicating that the genome structures of CRKP during the therapy are stable (Fig. 2A). Further genomic variants analysis showed accumulation of 4 mutations (including 1 SNP and 3 indels) in KP-2S, 25 mutations (including 12 SNPs and 13 indels) in KP-3R, and 20 mutations (including 11 SNPs and 9 indels) in KP-4R (Fig. 2B). Functional analysis of these mutations revealed that most of them are either synonymous mutations or located in intergenic regions. Based on the date of isolation of each strain, the spontaneous mutation rates were estimated for KP-2S, KP-3R, and KP-4R as 2.1 × 10^−6^, 1.8 × 10^−5^, and 1.5 × 10^−5^ substitutions per site per year, respectively (Fig. 2C).

To explore the genetic determinants associated with the phenotypic changes, mutations in KP-3R and KP-4R but not in KP-1S and KP-2S were identified (Table 2). A total of 14 variants were exclusively present in KP-3R and KP-4R, including 8 SNPs and 6 indels. Thirteen of fourteen variants were located in the intergenic regions, and the other was a 2,226 bp large deletion that contained the acyltransferase family protein-encoding gene *act*. The large deletion was located upstream of *ISKpn26*, an insertion sequence that commonly mediates DNA deletion in K. pneumoniae (Fig. S1). Eleven of thirteen intergenic variants were located far apart (>200 bp) from their adjacent genes and, thus, unlikely to contribute to phenotypic changes. Another mutation was a 12 bp deletion of the ribosomal binding site (RBS) of *ramR*, which had been reported to confer tigecycline resistance in our previous study. The impact of *ramR* on pathogenicity in K. pneumoniae is limited (13, 14). The last mutation is a 6 bp deletion (TGTTT) of the 42 bp upstream of *ompK26*. Therefore, the functions of TGTTT deletion upstream of *ompK26* and *act* deletion were the focus here.

**TABLE 2:** Genomic differences among the four ST11-CRKP isolates^*a*^

Contig.	POS	KP-1S	KP-2S	KP-3R	KP-4R	Annotation^*b*^
1	88,828	ATGTTT	ATGTTT	A	A	42 bp upstream of *ompK26* (GK022_00450)
8	158,433	T	T	G	G	Intergenic region of GK022_11360 and GK022_11365
14	29,796	G	G	C	C	Intergenic region of GK022_16760 and GK022_16765
14	29,933	GGA	GGA	CGC	CGC	Intergenic region of GK022_16760 and GK022_16765
14	29,975	CTTCGCTAAATGTG	CTTCGCTAAATGTG	GTTGTTATACGCAA	GTTGTTATACGCAA	Intergenic region of GK022_16760 and GK022_16765
14	29,993	G	G	A	A	Intergenic region of GK022_16760 and GK022_16765
14	30,013	T	T	C	C	Intergenic region of GK022_16760 and GK022_16765
14	30,020	A	A	G	G	Intergenic region of GK022_16760 and GK022_16765
27	9,749	AACCTGCGTGAGG	AACCTGCGTGAGG	A	A	9 bp upstream of *ramR* (GK022_23275)
43	1	T	T	DEL:1-2,226	DEL:1-2,226	*act* (GK022_27640), transposase (GK022_27645), small membrane protein (GK022_27650)
46	3,921	G	G	T	T	Intergenic region of GK022_28020 and GK022_28025
46	3,979	GTTGTTATACGCAAAAAAA	GTTGTTATACGCAAAAAAA	CTTCGCTAAATGTGAAAAG	CTTCGCTAAATGTGAAAAG	Intergenic region of GK022_28020 and GK022_28025
46	4,017	C	C	T	T	Intergenic region of GK022_28020 and GK022_28025
46	4,024	G	G	A	A	Intergenic region of GK022_28020 and GK022_28025

a Only the variants present in KP-3R and KP-4R but absent in KP-1S and KP-2S are shown.

b GK022_16760, GK022_16765, and GK022_28025 are hypothetical proteins with unknown function. GK022_11360 is annotated as 4-carboxymuconolactone decarboxylase. GK022_11365 is annotated as a mechanosensitive ion channel. GK022_28020 is annotated as AbrB/MazE/SpoVT family DNA-binding domain-containing protein.

10.1128/msystems.01342-21.5 FIG S1 Schematic diagram of the variants present in KP-3R and KP-4R. (A) 5 bp (TGTTT)-deletion found on 42 bp upstream of *ompK26*. (B) 2,226 bp large deletion located upstream of an insertion sequence *ISKpn26*. (C) 12 bp-deletion of *ramR* ribosomal binding site (RBS). Download FIG S1, PDF file, 0.1 MB. Copyright © 2022 Ye et al. 2022 Ye et al. https://creativecommons.org/licenses/by/4.0/ This content is distributed under the terms of the Creative Commons Attribution 4.0 International license.

### TGTTT deletion upregulated the expression of *ompK26* by disrupting the binding site of repressor KdgR and partially reduced the virulence of CRKP.

**FIG 3:**
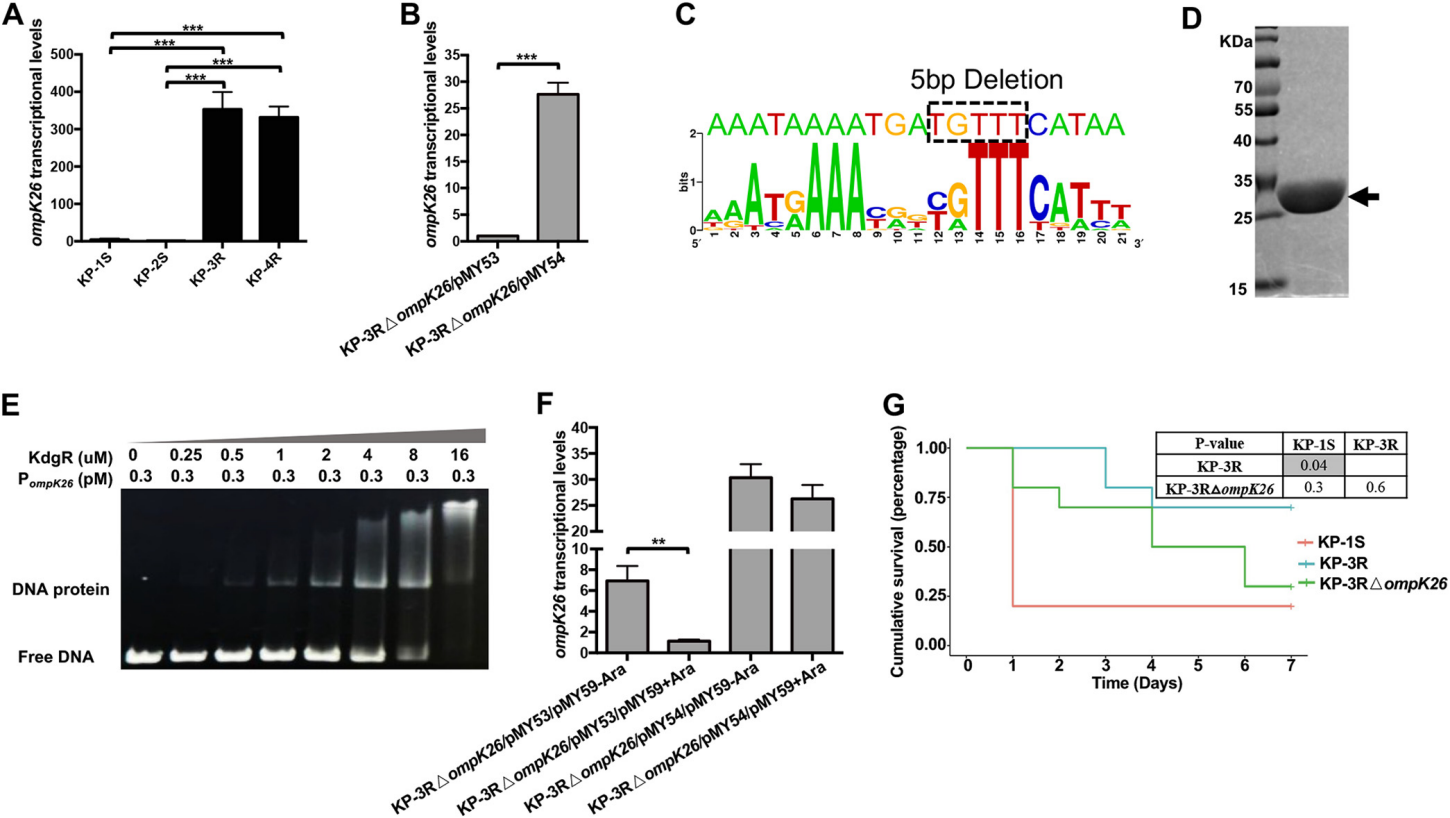
Functional study of *ompK26.* (A) Quantitative reverse transcription-PCR (qRT-PCR) assessment of the transcriptional level of *ompK26* in KP-1S, KP-2S, KP-3R, and KP-4R. (B) qRT-PCR assessment of the transcriptional level of *ompK26* in KP-3RΔ*ompK26*/pMY53 and KP-3RΔ*ompK26*/pMY54. (C) The prediction of KdgR binding site. The top sequence is the predicted KdgR binding site in the *ompK26* promoter. The bottom sequence is the conserved KdgR binding sequence. The 5 bp deletion sequence (dotted box) was located within the predicted KdgR binding site. (D) KdgR-6xHis protein following affinity purification. The arrow indicates recombinant KdgR protein. (E) EMSA using purified KdgR protein and 201 bp of promoter regions of *ompK26*. KdgR decreases the migration of promoter DNA of *ompK26*. (F) qRT-PCR assessment of the transcriptional level of *ompK26* in KP-3RΔ*ompK26*/pMY53/pMY59-Ara, KP-3RΔ*ompK26*/pMY53/pMY59+Ara, KP-3RΔ*ompK26*/pMY54/pMY59-Ara, and KP-3RΔ*ompK26*/pMY54/pMY59+Ara. (G) Kaplan-Meier survival curves of mice intraperitoneally challenged with KP-1S, KP-3R, and KP-3RΔ*ompK26*. Mice were inoculated with 10^6^ CFU and monitored for 7 days. *P* values were calculated using the Mantel-Cox log-rank test for survival curve comparison. Gray areas indicate significant values (<0.05). Other *P* values were calculated from Student's *t* test: *, *P* < 0.05; **, *P* < 0.01; ***, *P* < 0.001.

Given that TGTTT deletion was located 42 bp upstream of *ompK26*, we examined whether the deletion affected *ompK26* transcription in KP-3R and KP-4R. qRT-PCR analysis showed that transcriptional levels of *ompK26* in KP-3R and KP-4R were significantly higher (>300-fold; *P* < 0.001) than that in KP-1S and KP-2S (Fig. 3A). To further confirm the effect of TGTTT deletion on *ompK26* transcription, *ompK26* with its native promoter regions of KP-1S and KP-3R were cloned in a T-vector to generate pMY53 and pMY54 (Fig. S2) and transformed into KP-3RΔ*ompK26* (Fig. S3), respectively. As shown in Fig. 3B, the transcriptional level of *ompK26* in KP-3RΔ*ompK26*/pMY54, which harbors the TGTTT deletion, was significantly higher (>27-fold; *P* < 0.001) than that in KP-3RΔ*ompK26*/pMY53, which harbors the wild-type promoter, demonstrating that the deletion of TGTTT upregulated the *ompK26* expression.

10.1128/msystems.01342-21.6 FIG S2 Schematic diagram of the *ompK26* complementation plasmid pMY53 and pMY54. Download FIG S2, PDF file, 0.03 MB. Copyright © 2022 Ye et al. 2022 Ye et al. https://creativecommons.org/licenses/by/4.0/ This content is distributed under the terms of the Creative Commons Attribution 4.0 International license.

10.1128/msystems.01342-21.7 FIG S3 Construction of the *ompK26* and *act* mutant. (A and C) Strategy for constructing the *ompK26* and *act* mutant. Arrows indicate the positions of primers (labeled above the arrows) for PCR analyses. (B and D) PCR analysis of the wild-type and mutant strains. The specific primer pairs used in PCR are indicated at the top. Download FIG S3, PDF file, 0.4 MB. Copyright © 2022 Ye et al. 2022 Ye et al. https://creativecommons.org/licenses/by/4.0/ This content is distributed under the terms of the Creative Commons Attribution 4.0 International license.

Given that OmpK26 belongs to the KdgM family, its gene expression is often repressed by the repressor KdgR. Further sequence analysis identified a putative KdgR binding site upstream of *ompK26*, and that TGTTT deletion is within the binding region of KdgR (Fig. 3C). To determine if KdgR binds to the promoter region of *ompK26*, KdgR-6×His fusion protein was purified (Fig. 3D) and EMSA was performed. The results showed that KdgR can bind to the promoter region of *ompK26* (Fig. 3E). To further demonstrate that *ompK26* was regulated by KdgR in the cell and TGTTT deletion affects such regulation, KP-3RΔ*ompK26*/pMY53, and KP-3RΔ*ompK26*/pMY54 were complemented with a wild-type *kdgR* gene (pMY59) controlled by the arabinose-inducible promoter P_BAD_. As shown in Fig. 3F, when KdgR production was induced by arabinose, the transcriptional level of *ompK26* in KP-3RΔ*ompK26*/pMY53 (with wild-type promoter) was significantly repressed (>6-fold; *P* < 0.01). However, no repressive effect was observed in KP-3RΔ*ompK26*/pMY54 (harboring the TGTTT deletion). These results together demonstrated that KdgR represses *ompK26* expression, and TGTTT deletion upregulated the *ompK26* expression by affecting KdgR binding.

To investigate the role of OmpK26 in virulence, a mouse lethality study of KP-1S (low *ompK26* expression), KP-3R (*ompK26* overexpression), and KP-3RΔ*ompK26* (no *ompK26* expression) was conducted. Results showed that, although it was not statistically significant, there is a negative correlation between the mortality rate and the *ompK26* expression levels: high mortality of mice infected with no or low *ompK26* expression (KP-3RΔ*ompK26*, 70%; KP-1S, 80%), and low mortality with high *ompK26* expression (KP-3R, 30%) (Fig. 3G). These results suggested that *ompK26* may be associated with virulence of CRKP, and overexpression of *ompK26* may contribute to a reduced virulence phenotype observed in KP-3R.

### *act* is involved in the synthesis of CPS and deletion of *act* significantly attenuated the virulence of ST11-CRKP.

**FIG 4:**
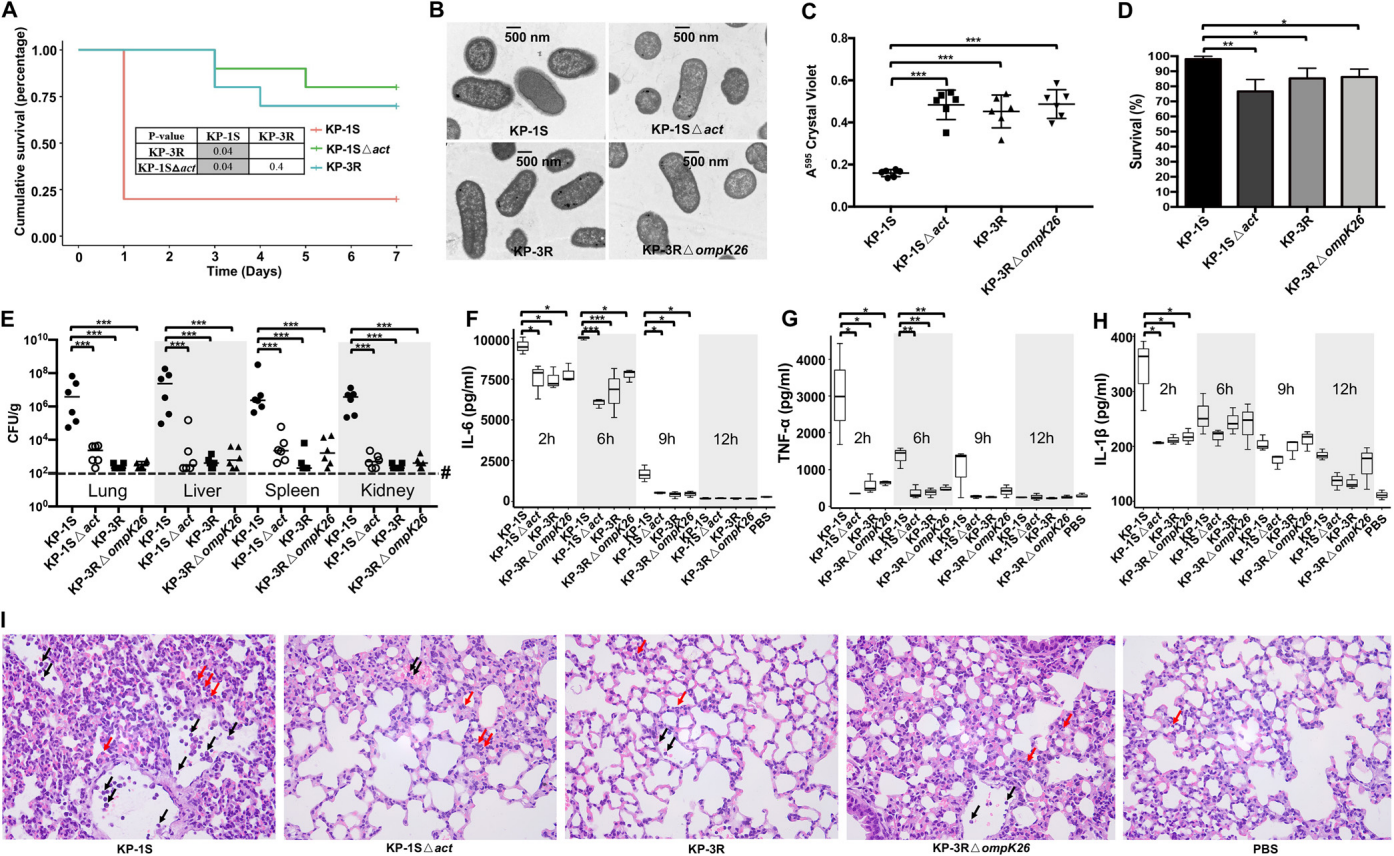
Functional study of *act*. (A) Kaplan-Meier survival curves of mice intraperitoneally challenged with KP-1S, KP-1S△*act*, and KP-3R. Mice were inoculated with 10^6^ CFU and monitored for 7 days. (B) Transmission electron microscopy showing the capsule morphology of KP-1S, KP-1SΔ*act*, KP-3R, and KP-3RΔ*ompK26*. One representative image from four images obtained from one section is shown. (C) Crystal violet assay for assessment of biofilm formation of KP-1S, KP-1SΔ*act*, KP-3R, and KP-3RΔ*ompK26*. (D) Survival of KP-1S, KP-1SΔ*act*, KP-3R, and KP-3RΔ*ompK26* within neutrophils. The survival index was calculated with the equation described in the method. (E) Bacterial loads of KP-1S, KP-1SΔ*act*, KP-3R, and KP-3RΔ*ompK26* in lung, liver, spleen, and kidney tissues. # denotes below the limit of detection. Horizontal lines represent median values, and each data point represents an individual mouse. (F to H) Selected cytokines in mouse serum quantified by ELISA. *n* = 3 per time point, Box-and-Whiskers with min/max presented. (I) Histopathology of the lung in mice infected with KP-1S, KP-1SΔ*act*, KP-3R, and KP-3RΔ*ompK26* (PBS as a control) in H&E-stained sections. Red arrows indicate infiltrated neutrophils in the alveolar wall, and black arrow indicates the neutrophils in the veins. *P* values were calculated using the Mantel-Cox log-rank test for survival curve comparison. Gray areas indicate significant values (<0.05). Other *P* values were calculated using Student's *t* test: *, *P* < 0.05; **, *P* < 0.01; ***, *P* < 0.001.

The function of the *act* gene in CRKP is unknown; however, it was colocalized with multiple genes involved in capsular production (UDP-glucose 6-dehydrogenase encoding gene *ugd* and UDP galacturonate 4-epimerase encoding gene *uge*). To study the role of *act* in pathogenesis, an *act* null mutant in KP-1S (KP-1SΔ*act*) was constructed by Lambda-Red (λ-Red) homologous recombination (Fig. S3). The infectivity was determined by intraperitoneally infecting mice with an inoculum of 10^6^ CFU. At 7 days postinfection (Fig. 4A), the survival rate of mice infected with KP-1SΔ*act* (80%) was significantly higher (*P* < 0.05) than that of mice infected with KP-1S (20%). No significant difference in survival rate was found between the groups of KP-1SΔ*act* and KP-3R. This result indicates that *act* deficiency reduces the virulence of ST11-CRKP, and 2,226 bp deletion containing *act* in KP-3R and KP-4R contributes a major part to the attenuation phenotype in these isolates.

To investigate the function of *act*, we further characterized the phenotypes of *act*-deficient mutants. First, the mucoviscosity was reduced in the KP-1SΔ*act* compared with KP-1S by the string test (Fig. S4). The transmission electron microscopy assay further showed that the KP-1SΔ*act* had reduced capsule production (Fig. 4B). Biofilm assay showed that the biofilm productions of KP-1SΔ*act*, KP-3R, and KP-3RΔ*ompK26* were significantly higher (*P* < 0.001) than that of KP-1S (Fig. 4C). The role of *act* in pathogenicity was evaluated by using human neutrophil killing assay and bacterial load analysis. As showed in Fig. 4D, the KP-1S had an average survival of 99.8% after incubation with human neutrophils for 60 min, which was significantly higher than that of KP-1SΔ*act* (76.6%, *P* < 0.01), KP-3R (85.3%, *P* < 0.05) and KP-3RΔ*ompK26* (86.2%, *P* < 0.05). The bacterial loads in the lung, liver, spleen, and kidney of infected mice were quantified following intraperitoneal inoculation of 10^7^ CFU throughout a 24 h infection. The results showed that the recovery of KP-1S from each tissue was significantly greater (*P* < 0.001) compared to KP-1SΔ*act*, KP-3R, and KP-3RΔ*ompK26* (Fig. 4E). These results indicate that *act* is involved in capsule production and biofilm formation and is important for preventing neutrophil killing and survival in host tissues of CRKP.

10.1128/msystems.01342-21.8 FIG S4 String test results of isolates plated on blood agar plates. Download FIG S4, PDF file, 1.3 MB. Copyright © 2022 Ye et al. 2022 Ye et al. https://creativecommons.org/licenses/by/4.0/ This content is distributed under the terms of the Creative Commons Attribution 4.0 International license.

To better understand the role of *act* in pathogenesis, inflammatory cytokine production throughout infection was quantified in mice infected with KP-1S, KP-1SΔ*act*, KP-3R, and KP-3RΔ*ompK26*. Initial induction of proinflammatory cytokines with high levels of IL-6, TNF-α, and IL-1β expression in serum by 2 h was observed in response to intraperitoneal inoculation with all isolates tested (Fig. 4F to H). However, the production of these proinflammatory cytokines quickly waned, and KP-1S induced the highest levels of these cytokines at all time points. The production of IL-6, TNF-α, and IL-1β were significantly higher (*P* < 0.05) in the serum of mice infected with KP-1S compared with mice infected with KP-1SΔ*act*, KP-3R, and KP-3RΔ*ompK26* at 2 h. Further histopathology of lung tissue showed that the alveolar wall was infiltrated with neutrophils and was thickened in mice infected with KP-1S, and a large number of neutrophils was observed in the veins (Fig. 4I). Compared with KP-1S, the inflammation was notably reduced in lung tissues of mice infected with KP-1SΔ*act*, KP-3R, and KP-3RΔ*ompK26*. In addition, more prominent inflammation in the liver, spleen, and kidney were observed with KP-1S infection compared to KP-1SΔ*act*, KP-3R, and KP-3RΔ*ompK26* (Fig. S5).

10.1128/msystems.01342-21.9 FIG S5 Microscopy of tissue sections stained with hematoxylin-eosin. Mice were intraperitoneally infected with 10^6^ CFUs of K. pneumoniae. Pathologic alterations of tissues were determined after 24 h of infection. In liver tissue infected with KP-1S, neutrophils (black arrow) can be seen in the central vein. Congestion and expansion of hepatic sinuses (yellow arrow) and neutrophil infiltration were observed (red arrow). In spleen tissue infected with KP-1S, a large amount of lymphocyte apoptosis (black arrow) and immature lymphocytes (red arrow) were observed. In kidney tissue infected with KP-1S, a large number of neutrophils (black arrows) can be seen in the vein. Compared with KP-1S, the inflammation was notably reduced in these tissues infected with KP-1SΔ*act*, KP-3R, and KP-3RΔ*ompK26*. Download FIG S5, PDF file, 0.6 MB. Copyright © 2022 Ye et al. 2022 Ye et al. https://creativecommons.org/licenses/by/4.0/ This content is distributed under the terms of the Creative Commons Attribution 4.0 International license.

### Transcriptome analysis identified genes associated with *act*.

**FIG 5:**
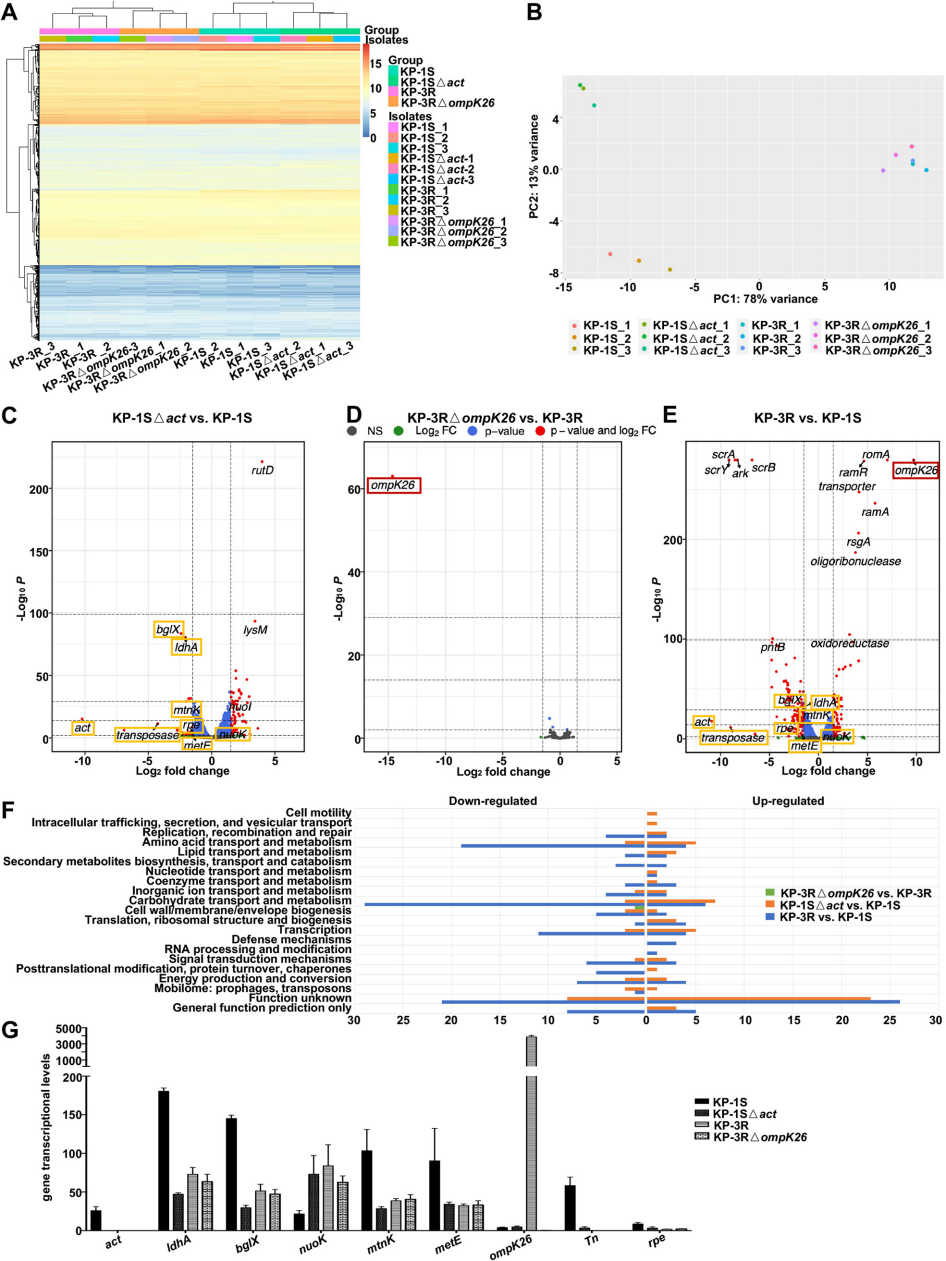
RNA-seq analysis of ST11-CRKP isolates. (A) The heatmap of transcriptional profiles of KP-1S, KP-3R, KP-1SΔ*act*, and KP-3RΔ*ompK26.* (B) Principal-component analysis (PCA) of transcriptional profiles of KP-1S, KP-3R, KP-1SΔ*act,* and KP-3RΔ*ompK26.* (C) The volcano plot of differentially expressed genes in KP-1SΔ*act* versus KP-1S. (D) The volcano plot of differentially expressed genes in KP-3RΔ*ompK26* versus KP-3R. (E) The volcano plot of differentially expressed genes in KP-3R versus KP-1S. Genes highlighted with rectangles are the differentially expressed genes shared among the compared groups. (F) COG analysis of the differentially expressed genes from each group. Genes with unknown functions were omitted. (G) The transcriptional levels of genes are differentially expressed in both compared groups KP-1SΔ*act* versus KP-1S and KP-3R versus KP-1S.

To explore how Act protein influences the functions observed above, we performed RNA-seq transcriptome analyses of KP-1S, KP-3R, KP-1SΔ*act*, and KP-3RΔ*ompK26* (Data Set S1). The heatmap showed that most of the genes were expressed uniformly across all the samples (Fig. 5A). The first principal component (PC1) and the second principal component (PC2) accounted for up to 91% of the variance of gene expression, indicating that only a few genes were differentially expressed and contributed to phenotype changes (Fig. 5B). The differentially expressed genes were identified between KP-1SΔ*act* and KP-1S, KP-3RΔ*ompK26* and KP-3R, as well as KP-3R and KP-1S (Data Set S1). When comparing the KP-1SΔ*act* and KP-1S, 21 genes were underexpressed and 61 genes were overexpressed in the KP-1SΔ*act*. In addition to *act*, other genes, including *ldhA*, *bglX*, *mtnK*, and *metE* were differentially expressed (Fig. 5C). When comparing KP-3RΔ*ompK26* and KP-3R, *ompK26* was the only gene showing under expressed in KP-3RΔ*ompK26* (Fig. 5D). When comparing KP-3R and KP-1S, 115 genes were underexpressed and 66 genes were overexpressed in KP-3R (Fig. 5E). In addition to *act* and *ompK26*, *ldhA*, *bglX*, *mtnK*, and *metE*, were also differentially expressed.

10.1128/msystems.01342-21.4 DATA SET S1 RNA-Seq analysis of KP-1S, KP-3R, KP-1S△act, and KP-3RΔompK26. Download Data Set S1, XLSX file, 2.6 MB. Copyright © 2022 Ye et al. 2022 Ye et al. https://creativecommons.org/licenses/by/4.0/ This content is distributed under the terms of the Creative Commons Attribution 4.0 International license.

Clusters of orthologous groups of protein (COG) functional analysis revealed that ‘cell wall/membrane/envelope biogenesis’ was affected by *ompK26* deletion (KP-3RΔ*ompK26* versus KP-3R) (Fig. 5F). The top three functions affected by *act* deletion (KP-1SΔ*act* versus KP-1S) were ‘carbohydrate transport and metabolism, ‘amino acid transport and metabolism, and ‘transcription’. Of note, though with additional genomic differences in KP-3R, the top three affected functions in KP-3R versus KP-1S were the same as those in KP-1SΔ*act* versus KP-1S. Given that differentially expressed genes presented simultaneously in KP-1SΔ*act* versus KP-1S and KP-3R versus KP-1S are more likely to be associated with the virulence phenotype, genes upregulated or downregulated in KP-1SΔ*act* and KP-3R compared with KP-1S were identified. Besides *act*, genes, including *ldhA*, *bglX*, *mtnK*, *metE*, transposon, and *rpe* were downregulated, and *nuoK* was upregulated in KP-1SΔ*act* and KP-3R (Fig. 5G). *ldhA* encodes d-Lactate dehydrogenase A and participated in fermentative lactate dehydrogenation. *bglX* encodes beta-glucosidase which hydrolyzes beta-d-glucosyl residues to beta-d-glucose. *mtnK* encodes S-methyl-5-thioribose kinase and *metE* encoded homocysteine S-methyltransferase. Both participated in the methionine synthase and methylation. *rpe* encodes ribulose-phosphate 3-epimerase catalyzes the reversible epimerization of d-ribulose 5-phosphate to d-xylulose 5-phosphate, which is important for carbohydrate degradation. *nuoK* (also known as ND4L) encodes NADH-quinone oxidoreductase subunit K and shuttles electrons from NADH to quinones in the respiratory chain. The results also showed that KP-3R yielded abundant transcripts of *ompK26*, suggesting that KdgR has a strong repressive effect on *ompK26*.

## DISCUSSION

In this study, the within-host genomic dynamics of ST11-CRKP during tigecycline therapy were deciphered with sequentially isolated ST11-CRKP strains from a patient with a scrotal abscess. The study begins with the ST11-CRKP strain that was initially susceptible then became resistant to tigecycline during tigecycline therapy. Tigecycline resistant ST11-CRKP infections are generally considered serious in clinical for their association with high mortality and poor outcomes (15). However, in this study, the patient dramatically recovered from the serious infection without effective medication treatment, and ST11-CRKP strains can be continually isolated from the urine samples of the patient. We found that the virulence of tigecycline resistant ST11-CRKP was attenuated compared to the initial tigecycline susceptible strain in the mice infection model, which restated the fitness cost theory of acquiring antibiotic resistance in K. pneumoniae.

Studies of pathogen adaptation during infection have been focused predominantly on mutations within coding regions, whereas adaptive mutations in intergenic regions received less attention (16, 17). The intergenic evolution might be a more cost-effective way than coding region evolution in the acquisition of novel phenotypes and mediating host adaptation. A previous study of intergenic evolution of Pseudomonas aeruginosa revealed that intergenic mutations play important roles in niche adaptation (18). In this study, almost all the mutations identified were located in the intergenic or regulatory regions, and two intergenic mutations that conferred host adaption were experimentally determined, which underlines the importance of intergenic evolution in within-host adaptation. Of note, the estimated spontaneous mutation rate of ST11-CRKP in this study was 2.1 × 10^−6^ to 1.8 × 10^−5^ substitutions per site per year, which is higher than the reported 6.9 × 10^−7^ to 1.8 × 10^−6^ substitutions per site per year (19, 20). The mutation rate might be overestimated in this study due to the limited sampling time points and the continuously selective pressure from antibiotics therapy.

The tigecycline resistance owing to the deletion of *ramR* RBS has been described in our previous publication (21). In this study, we investigated two additional mutations that relate to virulence attenuation phenotype upon antibiotic therapy, including the 5 bp (TGTTT)-deletion found upstream of *ompK26* and the deletion containing *act*. We demonstrated that the TGTTT deletion was located in the binding site of the repressor KdgR within the *ompK26* promoter, and the deletion increases the expression level of *ompK26* to a large extent. *ompK26* encodes a KdgM family porin and a knockout of *ompK26* has been reported to increase the virulence and carbapenem resistance in K. pneumoniae (22). Our results showed that knockout of *ompK26* slightly increases the virulence of CRKP despite the Mantel-Cox log-rank test showing no significance between the survival rates of KP-3RΔ*ompK26* and KP-3R infected mice. RNA-seq results did not reveal any differentially expressed gene. Thus, we conclude that *ompK26* might directly participate in the pathogenesis of K. pneumoniae. The *act* gene is predicted to encode an acyltransferase family protein and is colocalized with the genes involved in CPS synthesis. CPS presents on the surface of both Gram-positive and Gram-negative bacteria and is an important virulence factor mediating host immune response (23, 24). Acetylation of CPS is common in bacteria (25), and acetylation enhances antigenicity and immunogenicity of CPS in Escherichia coli (26), Streptococcus agalactiae (27), and Neisseria meningitidis (28). The KL64 CPS in K. pneumoniae has been reported to be acetylated (29). Therefore, we speculate that the CPS of our KL64-CRKP with the intact *act* is acetylated as well. Of note, removal of the gene encoding acyltransferase in the CPS synthesis region reduced acetylation and production of CPS in liver abscess-causing hvKP (30), whereas enhanced acetylation of CPS increases colony mucoviscosity and reduces biofilm formation in E. coli (31). In this study, we showed that the CPS production was reduced, biofilm formation was increased and the protection from CPS was weakened in both the *act* deletion isolate and the experimental *act* knockout strains, which may be ascribed to disruption of CPS acetylation medicated by *act*. However, the structure and monosaccharide components of CPS from *act* deficient strains remain to be furtherly determined. Acetylation of CPS increases the pathogenicity of K. pneumoniae type K57 by enhancing the immunoreactivity and increasing the induction of proinflammatory cytokines (32). In this study, higher induction of IL-6, TNF-α, and IL-1β and enhanced recruitment of neutrophils were observed in mice infected with *act* intact strains compared to the large *act* deletion strain or the *act* gene-specific knockout strain, indicating that the epitope or immunoreactivity of CPS in *act* deficient strains may be altered.

In the *act*-defective strains, the genes involved in lactate dehydrogenation (*ldhA*), beta-d-glucose synthesis (*bglX*), and methylation (*mtnK* and *metE*) were found underexpressed. Deletion of *ldhA* in N. meningitidis promotes biofilm formation (33). Thus, the increased biofilm formation observed in the *act*-defective strains might be a result of the low level of *ldhA* expression. As d-glucose synthesis and methylation are required for CPS biosynthesis, low expressions of *bglX*, *mtnK*, and *metE* also likely contributed to the decreased CPS productions in the *act*-defective strains. The functions of other differentially expressed genes in the *act*-defective strains, such as *rpe* and *nuoK*, in K. pneumoniae remain unclear and will be further explored in the future.

In summary, our work illustrated the within-host evolution of a worldwide-disseminated clone, ST11-CRKP, by leveraging the power of WGS in a combination of genetic and pathogenesis studies and established a connection between the genomic variants and host adaptation. Our results provide a better understanding of the evolutionary capacity and within-host adaptation of bacteria, which are important for developing strategies of pathogens surveillance and infection control in the future.

## MATERIALS AND METHODS

### Strains and antimicrobial susceptibility testing.

KP-1S, KP-2S, KP-3R, and KP-4R were isolated from the urine samples of a patient with scrotal abscess and urinary tract infection during antibiotics treatment. Detailed information of the patient and isolates were described in our previous work (21). Other strains used in this study were constructed from KP-1S and KP-3R. All strains were cultivated in a lysogeny broth (LB) medium at 37°C. Information of strains is indicated in Table S2. The MIC of antimicrobial agents was determined by using the CLSI reference broth microdilution method for K. pneumoniae clinical strains (34).

10.1128/msystems.01342-21.2 TABLE S2 Information of strains used in this study. Download Table S2, XLSX file, 0.01 MB. Copyright © 2022 Ye et al. 2022 Ye et al. https://creativecommons.org/licenses/by/4.0/ This content is distributed under the terms of the Creative Commons Attribution 4.0 International license.

### Survival rates.

Male ICR mice (6 to 8 weeks old, weighing 20 to 25g) were infected intraperitoneally with 10^6^ CFU of K. pneumoniae (10 mice/group). Mice were monitored for 7 days and assessed for death every 16 to 24 h. All animal experiments were performed following the protocols approved by the Animal Ethics Committee of Shanghai Skin Disease Hospital. All mouse experiments in this study were performed with bacteria grown overnight and subcultured to the logarithmic phase.

### Determination of bacterial loads from tissues and histopathology analysis.

Mice were infected intraperitoneally with 10^6^ CFU of K. pneumoniae (6 mice/group). After 24 h of infection, tissues were collected within 5 min of euthanasia. The lung, livers, kidneys, and spleens were each cut into two equal pieces. For bacterial load analysis, tissues were added an equal volume of PBS (100 μL PBS/100 mg tissue) and homogenized by a tissue homogenizer. Bacterial loads of each tissue were quantified by serial dilutions on LB agar plates. Experiments were done twice. For histopathological studies, tissues were fixed in 10% buffered formalin and subsequently embedded in paraffin. Serial 3 μm sections were stained with hematoxylin and eosin (H&E) to visualize tissue alterations.

### ELISA analysis.

Mice were infected intraperitoneally with 10^7^ CFU of K. pneumoniae (12 mice/group) and 100 μL of PBS (3 mice/group). After 2, 6, 9, and 12 h of infection, blood samples were collected and coagulated at room temperature for 20 min. Supernatants of blood samples were collected by centrifugation, and the concentrations of TNF-α, IL-6, and IL-1β in supernatants were determined by ELISA kits (NeoBioscience, Shenzhen, China) as per the manufacturer's protocols. Experiments were done twice.

### Human neutrophil assay.

The human neutrophil assay was performed as previously described (35). Briefly, neutrophils were collected from healthy volunteers with signed written consent. Polymorphprep™ (Axis-Shield) was used according to the manufacturer’s instructions to obtain neutrophils. Isolated neutrophils were adjusted to 1 × 10^7^ cell/mL in PBS and were used immediately. Neutrophils (1 × 10^6^) were precultured in flat-bottom plates for 30 min and incubated with 4 × 10^7^ CFU of opsonized K. pneumoniae. A test without neutrophils was set for each K. pneumoniae isolate as the positive control. At the end of the reaction, 0.1% Triton X-100 was added to lyse the neutrophils on ice for 15 min, and the reaction mixture was diluted and plated on LB agar. The bacterial survival rate was presented as the percentage of CFU (CFU of the experimental group divided by CFU of the control group).

### Whole-genome sequencing and bioinformatics analysis.

Genome sequencing was performed as described previously (36). Briefly, genomic DNA of KP-1S, KP-2S, KP-3R, and KP-4R were extracted using a bacterial genomic DNA extraction kit and sequenced using Illumina HiSeq 150 bp paired-end sequencing technologies. The sequencing reads were assembled using SPAdes V3.8 (37) with default parameters and contigs with less than 500 nucleotides were excluded. The genes were predicted and annotated using NCBI online annotation service. Genome alignments were performed by MUMmer 4 (38). The SNPs and indels were identified by Snippy v4.6 and CNOGpro with KP-1S as the reference. The spontaneous mutation rate was determined by dividing the root-to-tip number of mutations by the days of strain isolation. The phylogenetic relationship was constructed by the FastTree (39) based on the variants with the maximum-parsimony method. The genomic features, including sequence typing, virulence genes, antimicrobial resistance genes, MLST, and capsular type were analyzed by Kleborate v2.0.1. Plasmid replicons were identified by PlasmidFinder.

### Quantitative RT-PCR (qRT-PCR).

qRT-PCR was performed as described previously (21). Briefly, RNA was extracted from mid-log-phase bacterial cultures using the RNeasy Minikit (Qiagen). cDNA was synthesized using the RT reagent kit with a gDNA Eraser (TaKaRa). qRT-PCR was performed using SYBR Premix *Ex Taq* (TaKaRa) on a CFX96 Real-Time PCR Detection System (Bio-Rad). PCR primers for *ompK26* and the endogenous reference gene *rrsE* were provided in Table S3.

10.1128/msystems.01342-21.3 TABLE S3 Primers used in this study. Download Table S3, XLSX file, 0.01 MB. Copyright © 2022 Ye et al. 2022 Ye et al. https://creativecommons.org/licenses/by/4.0/ This content is distributed under the terms of the Creative Commons Attribution 4.0 International license.

### Construction of *ompK26* and *act* mutant.

Knockout of chromosomal *ompK26* and *act* was conducted as we described previously (40). Briefly, pKOBEG was transformed into KP-3R to generate Kp-3R/pKOBEG. Homology fragments of *ompK26* were amplified and inserted into the pMD-18T-hph on either side of the hygromycin gene. The recombinant plasmid was then digested by KpnI and HindIII to get the final linear fragment. The final fragment was transformed into Kp-3R/pKOBEG. Mutant clones were screened by PCR using the primer pairs of internal-F/internal-R and external-F/external-R. The same method was applied to construct *act* mutants. Strategy for constructing and identification of mutant clones are shown in Fig. S3.

### Complementation of *ompK26* with its native promoter and *kdgR* overexpression.

The *ompK26* and the native promoter region of KP-1S and KP-3R were amplified by *ompK26*_promoter_F/R (Table S3) and cloned into pMD-18T-hph at HindIII and KpnI sites to generate pMY53 and pMY54, respectively. pMY53 and pMY54 were electrically transformed into KP-3RΔ*ompK26* to generate KP-3RΔ*ompK26*/pMY53 and KP-3RΔ*ompK26*/pMY54. Strategies for construction pMY53 and pMY54 are shown in Fig. S2. The full-length *kdgR* was amplified from KP-1S using pBAD33_*kdgR*_F/R (Table S3) and cloned into pBAD33 to generate pMY59. pMY59 was then transformed into KP-3RΔ*ompK26*/pMY53 and KP-3RΔ*ompK26*/pMY54. KP-3RΔ*ompK26*/pMY53/pMY59 and KP-3RΔ*ompK26*/pMY54/pMY59 were confirmed by qRT-PCR.

### Purification of recombinant KdgR and electrophoretic mobility shift assay (EMSA).

The full-length *kdgR* was amplified from KP-1S using pET28a_*kdgR*_F/R (Table S3) and cloned into pET-28a to generate pMY55. The KdgR-6×His fusion protein was expressed in BL21(DE3) with 0.2 mM isopropyl β-d-1-thiogalactopyranoside at 18°C. Protein purification was performed as previously described (21). Protein purity was confirmed by sodium dodecyl sulfate-polyacrylamide gel electrophoresis (SDS-PAGE) analysis. The 201 bp upstream of *ompK26* promoter regions were amplified using *ompK26*_prob_F/R (Table S3). The KdgR/DNA complexes were mixed, incubated, electrophoresis, and imaged according to the procedure described previously (41).

### Biofilm formation and transmission electron microscopy (TEM).

Biofilm production was determined as described (42). Briefly, 1 μL of overnight culture was inoculated into 100 μL of fresh LB broth in each well of untreated 96-well polystyrene plates. After 24 h of incubation at 37°C, the wells were washed four times with water and 150 μL of 0.1% crystal violet was added. After 10 min incubation, crystal violet was removed, and the wells were washed six times with water. Then, 200 μL of 80% ethanol was added and the plate was incubated for 10 min at room temperature before determining the OD595 with a microplate reader. Transmission electron microscopy was performed by the Electron Microscopy Facility of Servicebio (Wuhan, China), and images were captured by HITACHI HT7800/HT7700.

### RNA sequencing and differential expression analysis.

Overnight cultures of strains were diluted 1:100 in LB and cultured at 37°C with shaking to reach the mid-log-phase. RNA was extracted from bacterial cultures using the RNeasy Minikit (Qiagen). Total RNA was used as input material for the RNA sample preparations. RNA sequencing libraries were prepared according to the manufacturer's protocol and sequenced on the Illumina Novaseq platform. The genome and gene model annotation of KP-1S was used as the reference. The reads were mapped to the reference genome by Bowtie2 v2.4.2 (43). Differential expression analysis of two conditions/groups (three biological replicates per condition) was performed using DESeq (1.18.0) (44). *P* values were adjusted using Benjamini and Hochberg’s approach. Genes with adjusted *P* value <0.001 and |log_2_ change| >1.5 were classified as differentially expressed. Clusters of orthologous groups of proteins (COGs) database were used to classify the differentially expressed genes.

### Statistical analysis.

Statistical analysis was performed by R. The Mantel-Cox log-rank test was used to compare the Kaplan-Meier survival curves and calculate the *P* values. A Student's *t* test was used to compare the biofilm productions and calculate the *P* values.

### Data availability.

The genome sequences of KP-1S, KP-2S, KP-3R, and KP-4R, and raw RNA-seq data of KP-1S, KP-1SΔ*act*, KP-3R, and KP-3RΔ*ompK26* have been deposited in the DDBJ/ENA/GenBank under the bioproject accession number PRJNA590579.
