# Erratum for Ye et al., “Reduced Virulence and Enhanced Host Adaption during Antibiotics Therapy: a Story of a Within-Host Carbapenem-Resistant Klebsiella pneumoniae Sequence Type 11 Evolution in a Patient with a Serious Scrotal Abscess”

**DOI:** 10.1128/msystems.00545-22

**Published:** 2022-06-23

**Authors:** Meiping Ye, Chunjie Liao, Mengya Shang, Danyang Zou, Xin Feng, Xinying Lu, Yixin Zhang, Jingmin Yan, Zhixiang Hu, Xiaogang Xu, Jianping Jiang, Pingyu Zhou

**Affiliations:** a STD Institute, Shanghai Skin Disease Hospital, Tongji University School of Medicine, Shanghai, China; b Shanghai Skin Disease Hospital, Clinical School of Anhui Medical University, Shanghai, China; c Institute of Antibiotics, Huashan Hospital, Fudan University, Shanghai, China

## ERRATUM

Volume 7, no. 2, 2022, e01342-21, https://journals.asm.org/doi/10.1128/msystems.01342-21. Page 1: The gray box incorrectly lists Jianping Jiang’s email address as zhoupingyu@medmail.com.cn. It should be jiangjianping@fudan.edu.cn. In addition, this paper was originally posted on 23 February 2022, not 1 March 2022.

