# Supplementary figures and images for: Reduced Virulence and Enhanced Host Adaption during Antibiotics Therapy: a Story of a Within-Host Carbapenem-Resistant Klebsiella pneumoniae Sequence Type 11 Evolution in a Patient with a Serious Scrotal Abscess
Source: mSystems. 2022 Mar 1;7(2):e01342-21. doi: 10.1128/msystems.01342-21 (PMC9040587; doi:10.1128/msystems.01342-21)

**A**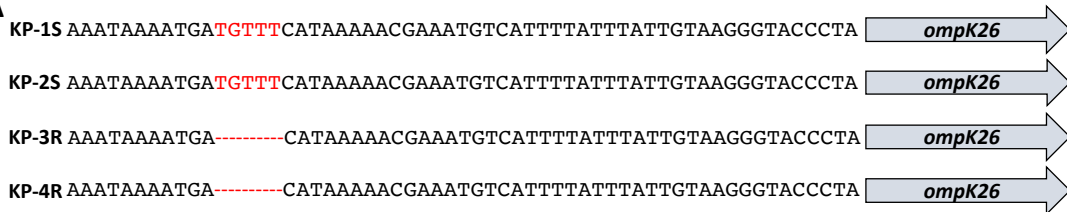**B**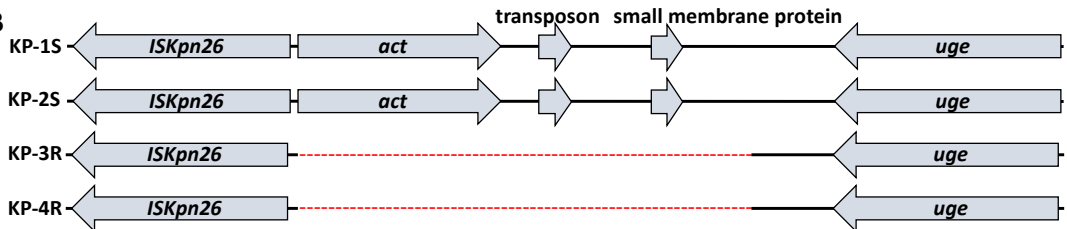**C**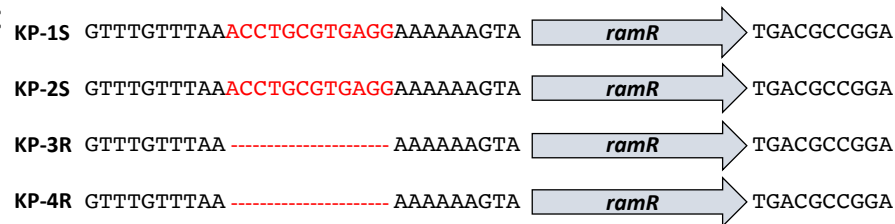

Supplement: FIG S1 [file msystems.01342-21-sf001.pdf]

**A**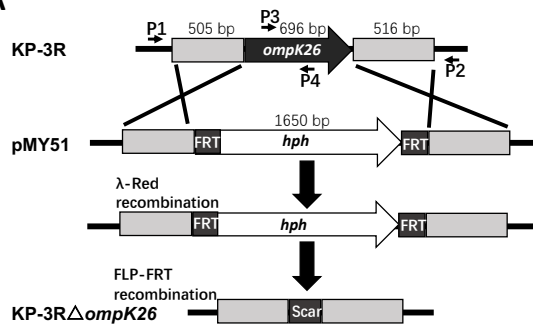**B**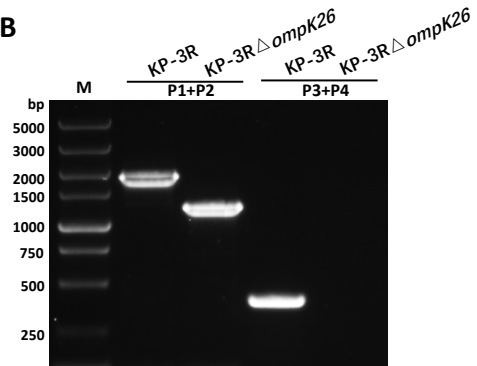**C**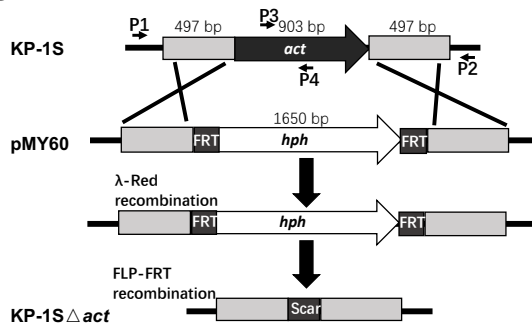**D**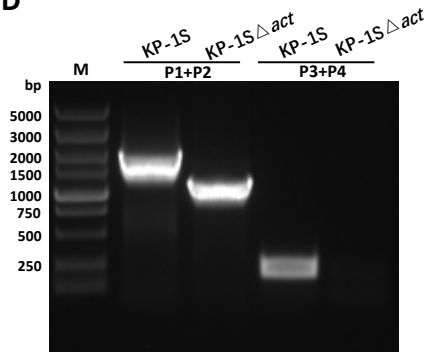

Supplement: FIG S3 [file msystems.01342-21-sf003.pdf]

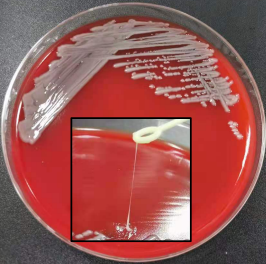

**KP-1S**

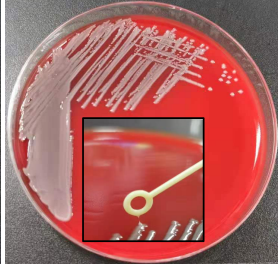

**KP-1S  $\Delta$  *act***

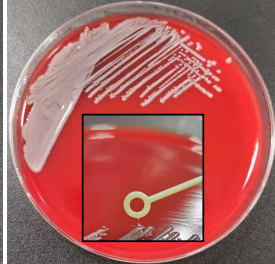

**KP-3R**

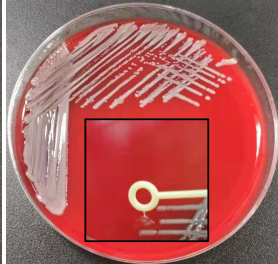

**KP-3R  $\Delta$  *ompK26***

Supplement: FIG S4 [file msystems.01342-21-sf004.pdf]

Liver

KP-1S

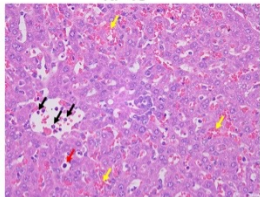

KP-1S $\Delta act$

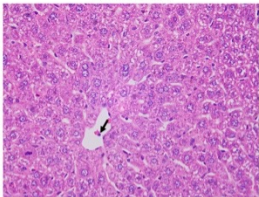

KP-3R

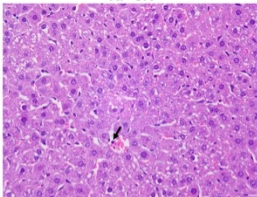

KP-3R $\Delta ompK26$

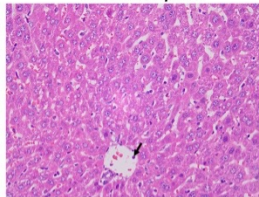

PBS

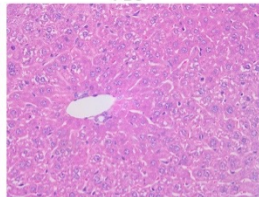

Spleen

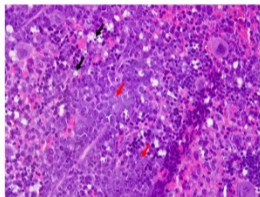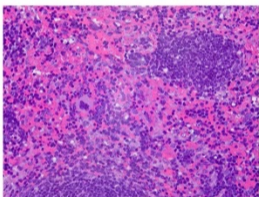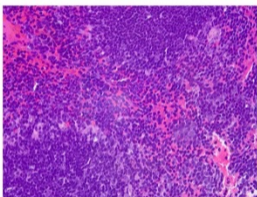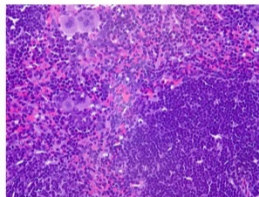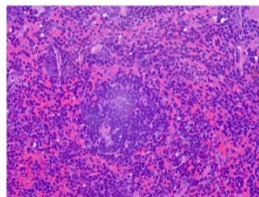

Kidney

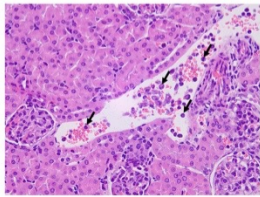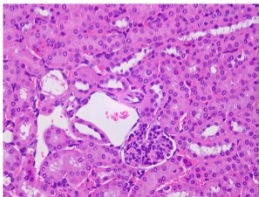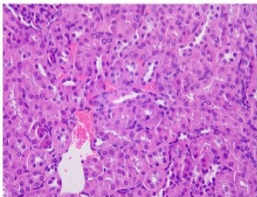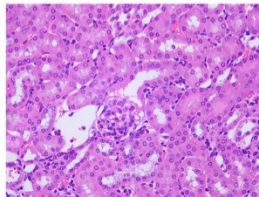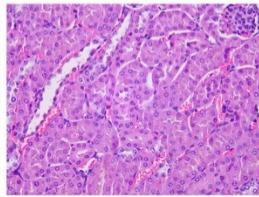

Supplement: FIG S5 [file msystems.01342-21-sf005.pdf]
