# Supplementary material for: Reduced Virulence and Enhanced Host Adaption during Antibiotics Therapy: a Story of a Within-Host Carbapenem-Resistant Klebsiella pneumoniae Sequence Type 11 Evolution in a Patient with a Serious Scrotal Abscess
Source: mSystems. 2022 Mar 1;7(2):e01342-21. doi: 10.1128/msystems.01342-21 (PMC9040587; doi:10.1128/msystems.01342-21)
Supplement: FIG S2 [file msystems.01342-21-sf002.pdf]

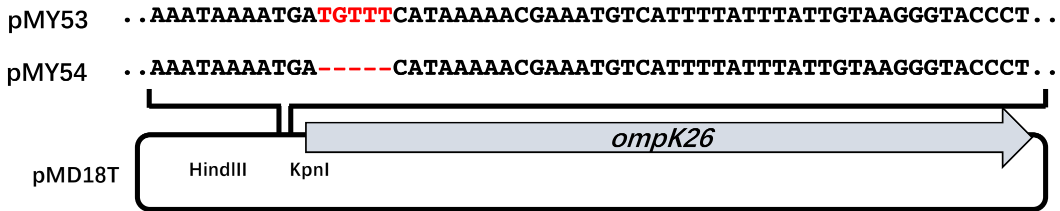

pMY53 ..AAATAAAATGATGTTTCATAAAAACGAAATGTCATTTTATTTATTGTAAGGGTACCCT..
pMY54 ..AAATAAAATGA-----CATAAAAACGAAATGTCATTTTATTTATTGTAAGGGTACCCT..
ompK26
pMD18T
HindIII
KpnI
